# Knowledge mapping of idiopathic scoliosis genes and research hotspots (2002–2022): a bibliometric analysis

**DOI:** 10.3389/fped.2023.1177983

**Published:** 2023-12-04

**Authors:** Like Ru, Hong Zheng, Wenjun Lian, Shuying Zhao, Qimeng Fan

**Affiliations:** ^1^ The First Affiliated Hospital of Henan University of Chinese Medicine, Zhengzhou, China; ^2^ School of Pediatrics, Henan University of Chinese Medicine, Zhengzhou, China

**Keywords:** idiopathic scoliosis, genes, data visualization, bibliometrics, Citespace, VOSviewer

## Abstract

**Background:**

The etiology of idiopathic scoliosis (IS) remains unclear. Gene-based studies on genetic etiology and molecular mechanisms have improved our understanding of IS and guided treatment and diagnosis. Therefore, it is imperative to explicate and demarcate the preponderant areas of inquiry, key scholars, and their aggregate scholarly output, in addition to the collaborative associations amongst publications or researchers.

**Methods:**

Documents were retrieved from the Web of Science Core Collection (WoSCC) with the following criteria: TS = (“idiopathic scoliosis” AND gene) refined by search operators (genomic OR “hereditary substance” OR “germ plasm” OR Cistrons OR genetics OR genetic OR genes OR Polygenic OR genotype OR genome OR allele OR polygenes OR Polygene) AND DOCUMENT TYPES (ARTICLE OR REVIEW), and the timespan of 2002-01-01 to 2022-11-26. The online bibliometric analysis platform (bibliometric), bibliographic item co-occurrence matrix builder (BICOMB), CiteSpace 6.1. R6 and VOS viewer were used to evaluate articles for publications, nations, institutions, journals, references, knowledge bases, keywords, and research hotspots.

**Results:**

A total of 479 documents were retrieved from WoSCC. Fourty-four countries published relevant articles. The country with the most significant number of articles was China, and the institution with the most significant number of articles was Nanjing University. Citation analysis formed eight meaningful clusters and 16 high-frequency keywords. (2) The citation knowledge map included single nucleotide polymorphisms, whole exome sequencing, axonal dynamin, drug development, mesenchymal stem cells, dietary intake, curve progression, zebrafish development model, extracellular matrix, and rare variants were the current research hotspots and frontiers.

**Conclusions:**

Recent research has focused on IS-related genes, whereas the extracellular matrix and unusual variants are research frontiers and hotspots. Functional analysis of susceptibility genes will prove to be valuable for identifying this disease.

## 1. Introduction

Idiopathic scoliosis (IS) is a three-dimensional spinal deformity in the coronal, sagittal, and horizontal planes that occurs without underlying vertebral abnormalities or significant physiological defects. The pathogenesis of IS remains unknown and poses a considerable challenge to its prevention and treatment (1–3). Human genetic studies have revealed the existence of complex polygenic diseases with genetics and phenotypes (4–6). The consistency of IS estimates ranged from 0.13 to 0.73 with monozygotic twins and from 0.00 to 0.36 with dizygotic twins (7). Autosomal dominant inheritance, x-linked dominant inheritance, and autosomal recessive inheritance models have been reported in the literature (8). IS is significantly associated with Single Nucleotide Polymorphism (SNPs) in several genes in multiple study populations, such as Melatonin receptor 1B (*MTNR1B*), Estrogen receptor1 (*ESR1*), and Matrilin 1 (*MATN1*) (9). Despite the vast amount of research conducted on IS-related genes, the specific mechanism underlying altered spine anatomy remains elusive (10). Several types of pathogenic gene mutations have been identified in idiopathic scoliosis through genetic studies, including a group of the *TGFB1* gene and the *MiR4300HG* gene that are related to the severity of the curve; a category of *LBX1*, *NTF3* and *SOCS3* that can predict progress curves; and a cohort of susceptibility loci in *SOX9*, *MATN1*, *AJAP1*, *MMP9* and *TIMP2* associated with cartilage and extracellular matrix (11, 12).

Based on these findings, idiopathic scoliosis has been extensively studied genetically, particularly regarding potential pathogenic mechanisms and susceptibility genes. However, it is unclear how the current research situation has developed. Bibliometric analysis is a method that employs objective statistical analysis to quantify the data characteristics and temporal trends of scientific literature pertinent to a given research project (13). Compared with traditional expert-written systematic reviews, bibliometric analyses provide a timely, intuitive, and unbiased way to track developments and explore specific areas of knowledge. In this study, we investigated Science Citation Index Expanded (SCIE) papers on IS-related gene studies and established a visual knowledge map of the area based on the cited references. Furthermore, we identified research hotspots using burst keywords. The above investigations provide researchers with a macro- and micro-level understanding of the entire field of knowledge.

## 2. Methods

### 2.1. Database

To ensure the stability of the search results, all data were downloaded from the Web of Science Core Collection (WoSCC) on Nov 26, 2022.

### 2.2. Search strategy

Based on the keyword search strategy, the search was constructed using keywords such as "idiopathic scoliosis" and "gene", and the types of filtered articles included "articles" and "reviews'. By limiting the search period to Nov 26, 2022, two researchers (LR and WL) independently confirmed the results. Detailed search strategies are presented in Table 1.

**Table 1:** The search strategy summary.

Items	Specification
Date of Search	Nov 26, 2022 11:05:00 GMT+0800 (China standard time)
Database	Web of Science Core Collection
Search strategy	# 1: ((((((((((((((TS=(genomic)) OR TS = (“hereditary substance”)) OR TS = (“germ plasm”)) OR TS = (Cistrons)) OR TS = (Cistron)) OR TS = (genetics)) OR TS = (genetic)) OR TS = (gene)) OR TS = (genes)) OR TS = (Polygenic)) OR TS = (genotype)) OR TS = (genome)) OR TS = (allele)) OR TS = (Polygenes)) OR TS = (Polygene) #2: TS = (“idiopathic scoliosis”) #3: #2 AND #1 and articles or reviews (Document types)
Time limit	2002-01-01 to 2022-11-26
Retrieved result	506

### 2.3. Data collection

**Figure 1:**
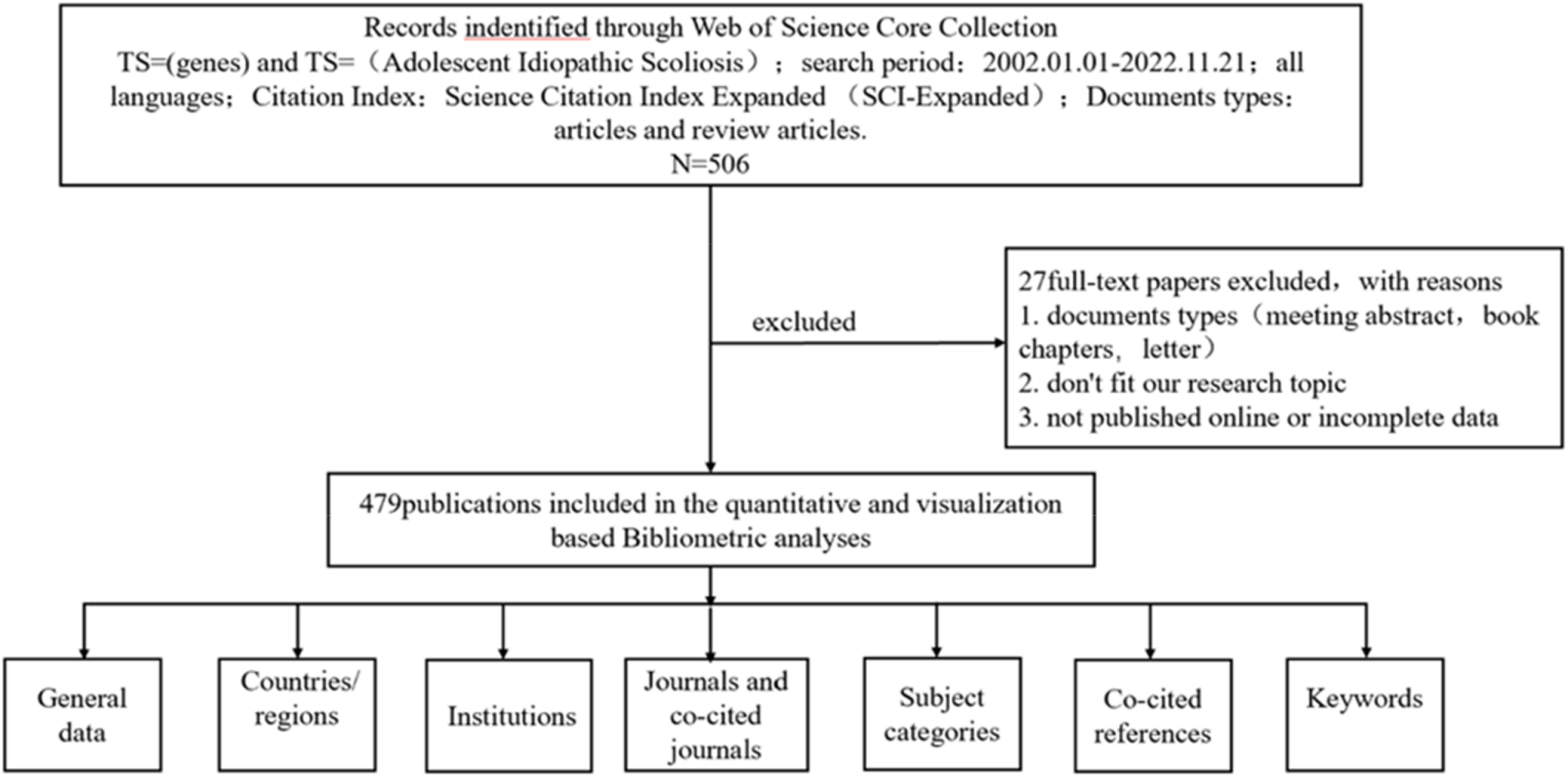
A frame flow diagram. The diagram displayed details selection criteria for Idiopathic Scoliosis gene publications from the WoSCC database and the steps of bibliometric analysis.

After completing the literature search, we exported the results to a complete recording format for analysis. We collected the following fundamental data for each article: title, abstract, authors, organization, nation, journal, keywords, and references. Two researchers (LR and WL) manually screened the imported data source files using EndNote. The inclusion criteria for the literature are as follows: (1) The literature must be a study on genes or gene mutations related to human idiopathic scoliosis; (2) The publication date must be between January 1, 2002 and November 26, 2022; (3) The article must be published in the SCIE database and have a certain degree of academic authority; (4) The literature must be of high-quality research types, such as original research, review, meta-analysis, clinical trial, systematic evaluation, etc.; (5) The literature must have reliability and reproducibility, including clear research objectives, methods, results, conclusions, and sufficient data support. Exclusion criteria include: (1) Articles that are not related to genes or gene mutations related to human idiopathic scoliosis; (2) Conference abstracts, book chapters, data papers, editorial materials, and reproductions that are unrelated to the topic; (3) Publications that were published earlier than 2002 or after November 26, 2022, or unpublished documents that cannot obtain full-text or do not have enough information for further analysis. Finally, 27 articles were excluded (Figure 1).

### 2.4. Data analysis

All aspects of publications were analyzed, including countries, institutions, journals, and keywords. An online bibliometric analysis platform (http://bibliometric.com/) and VOSviewer (University Leiden Netherlands) were used for co-occurrence analysis, and the results were displayed using collaborative network maps. The basic version of Citespace 6.1. R6 software (14) was used to analyze journals, references, and keywords. We set the operating parameters to # Years Per Slice = 1, TopN = 100, Top N% = 5%, and K = 10 by using "T" button to cluster and label the reference, after which the centrality of the nodes was calculated. The most-cited articles are then displayed.

## 3. Results

### 3.1. Distribution of literature by year of publication

**Figure 2:**
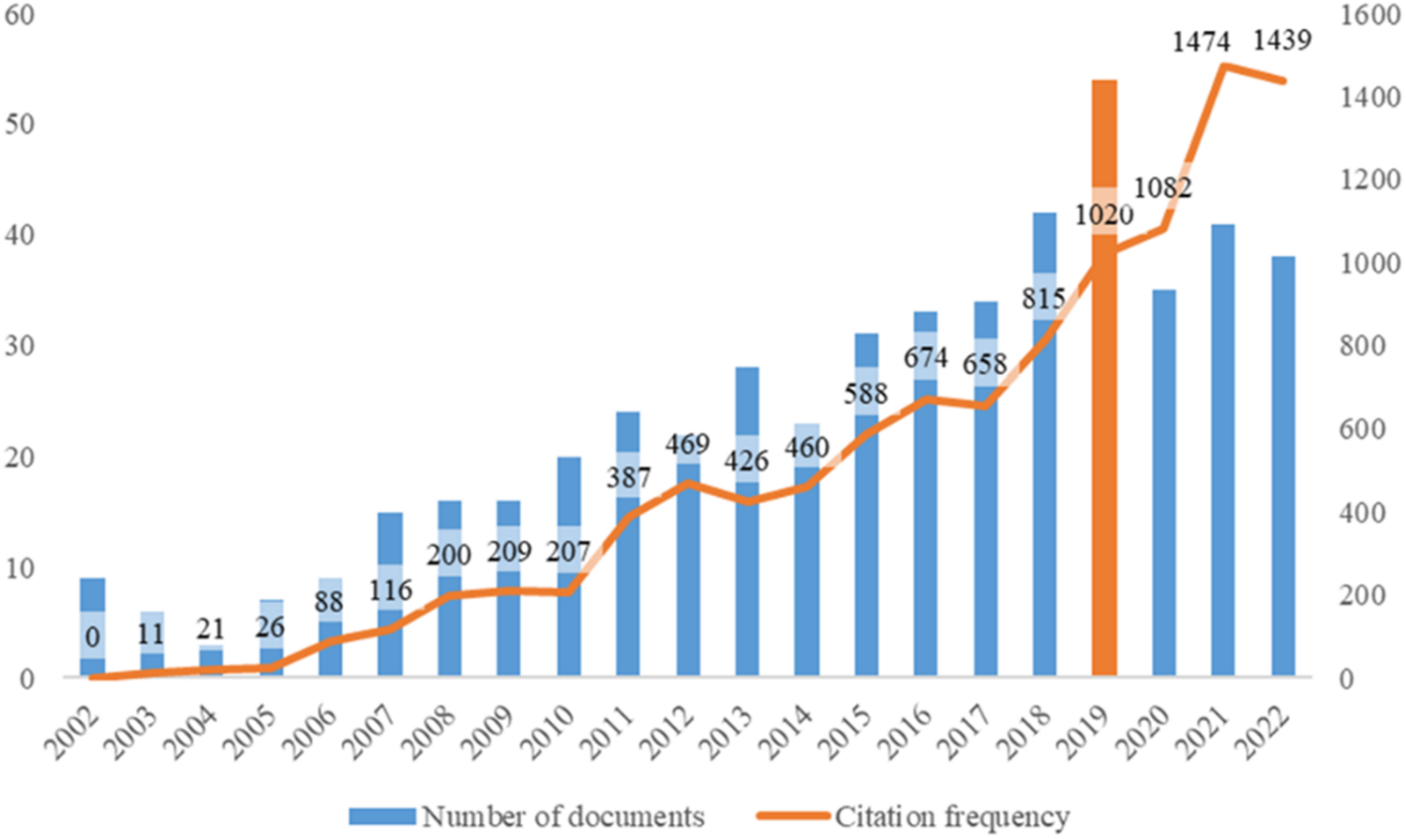
Number of citations of journal publications.

A total of 479 items were included in this topic from 2002 to Nov 26, 2022. Idiopathic scoliosis gene studies peaked in 2019, and the frequency peaked in 2021 (Figure 2).

### 3.2. Countries/regions and institutions

**Figure 3:**
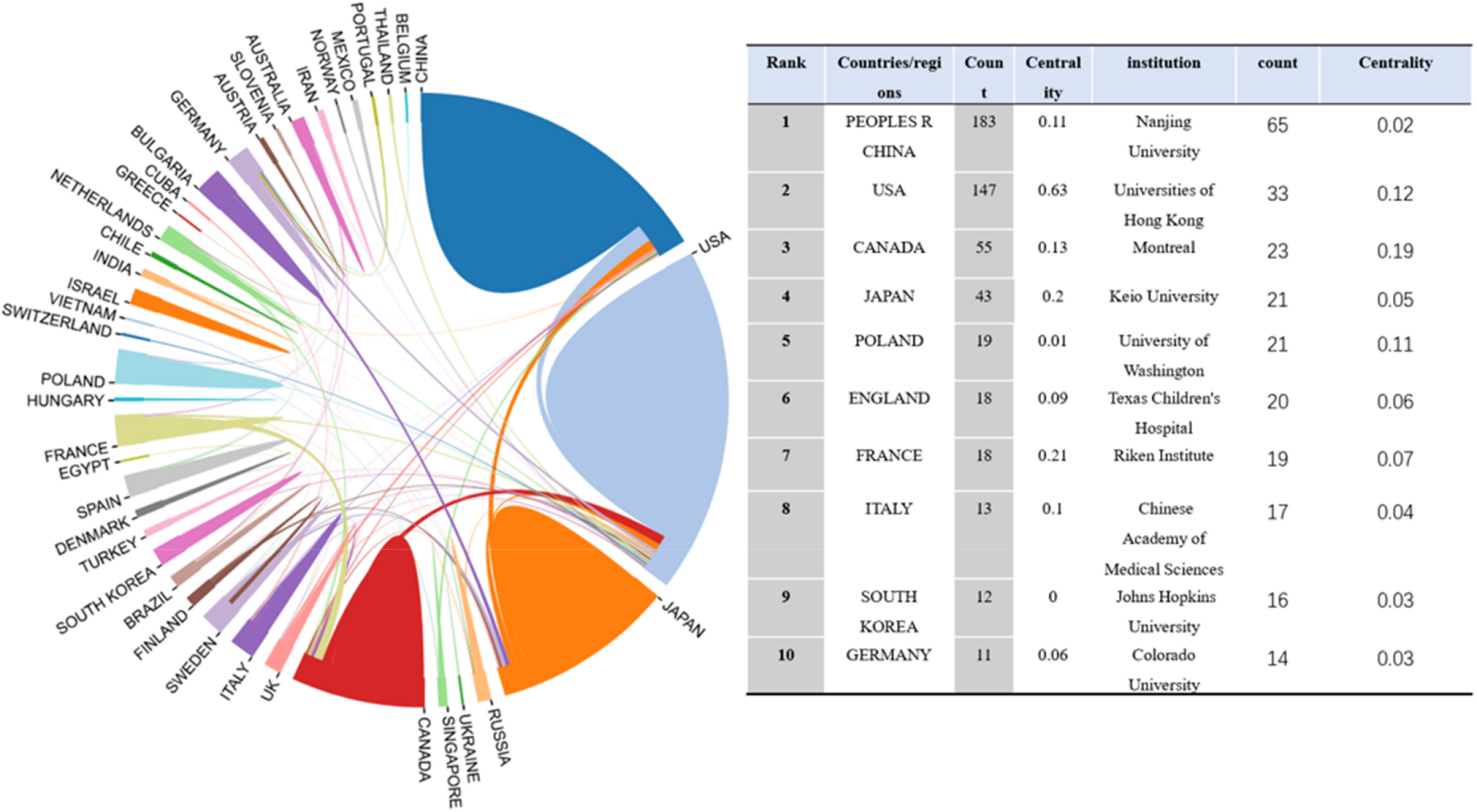
Top 10 countries and institutions for iS gene research (2002–2022).

**Figure 4:**
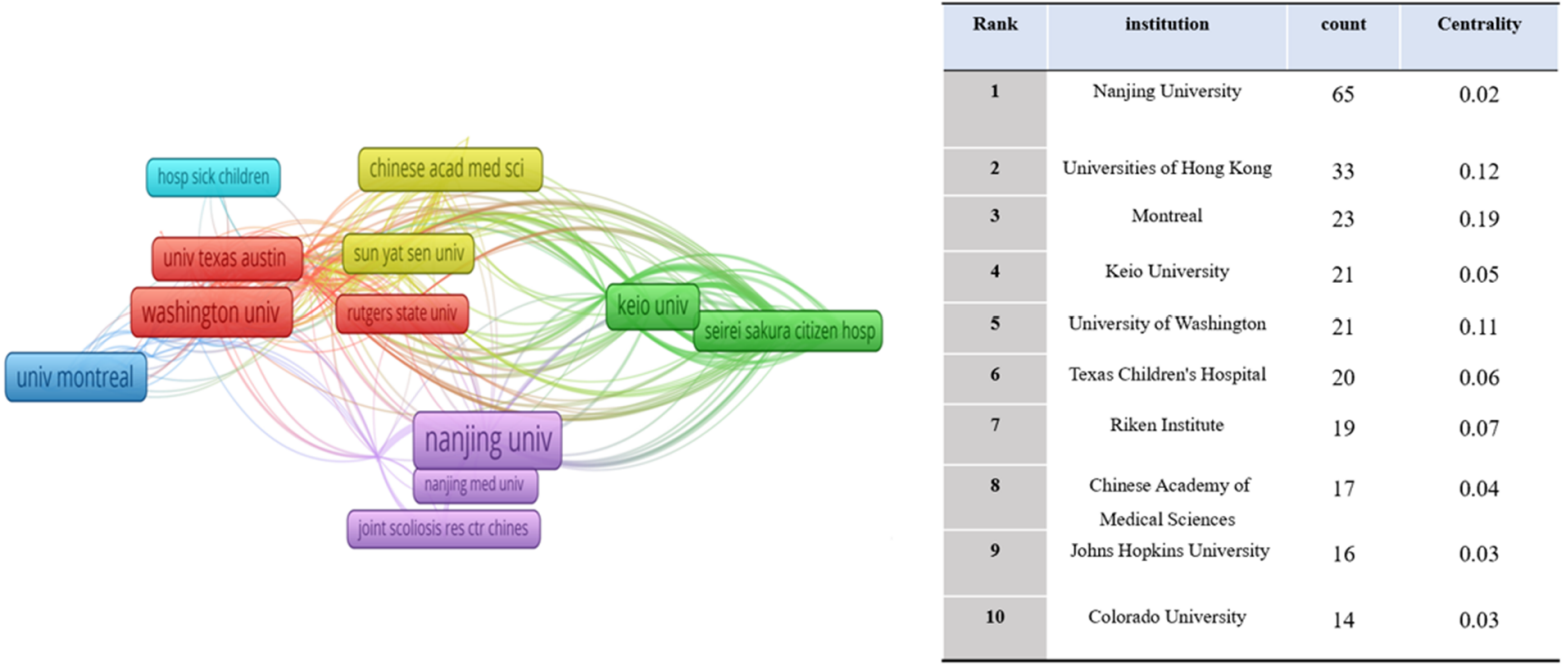
Network map of institutions contributed to publications on idiopathic scoliosis-genes from 2002 to 2022.

Between 2002 and Nov 26, 2022, studies on idiopathic scoliosis gene research were published by 44 nations/regions, and these nations/regions cooperated. China published the most articles (183, 38.2%), followed by the United States (147, 30.6%), Canada (55), and Japan (43) Figure 3). Most publications were published by Nanjing University (65), followed by Universities of Hong Kong (33), Montreal (23), and Keio University (21) (Figure 4).

### 3.3. Analysis of journals

**Figure 5:**
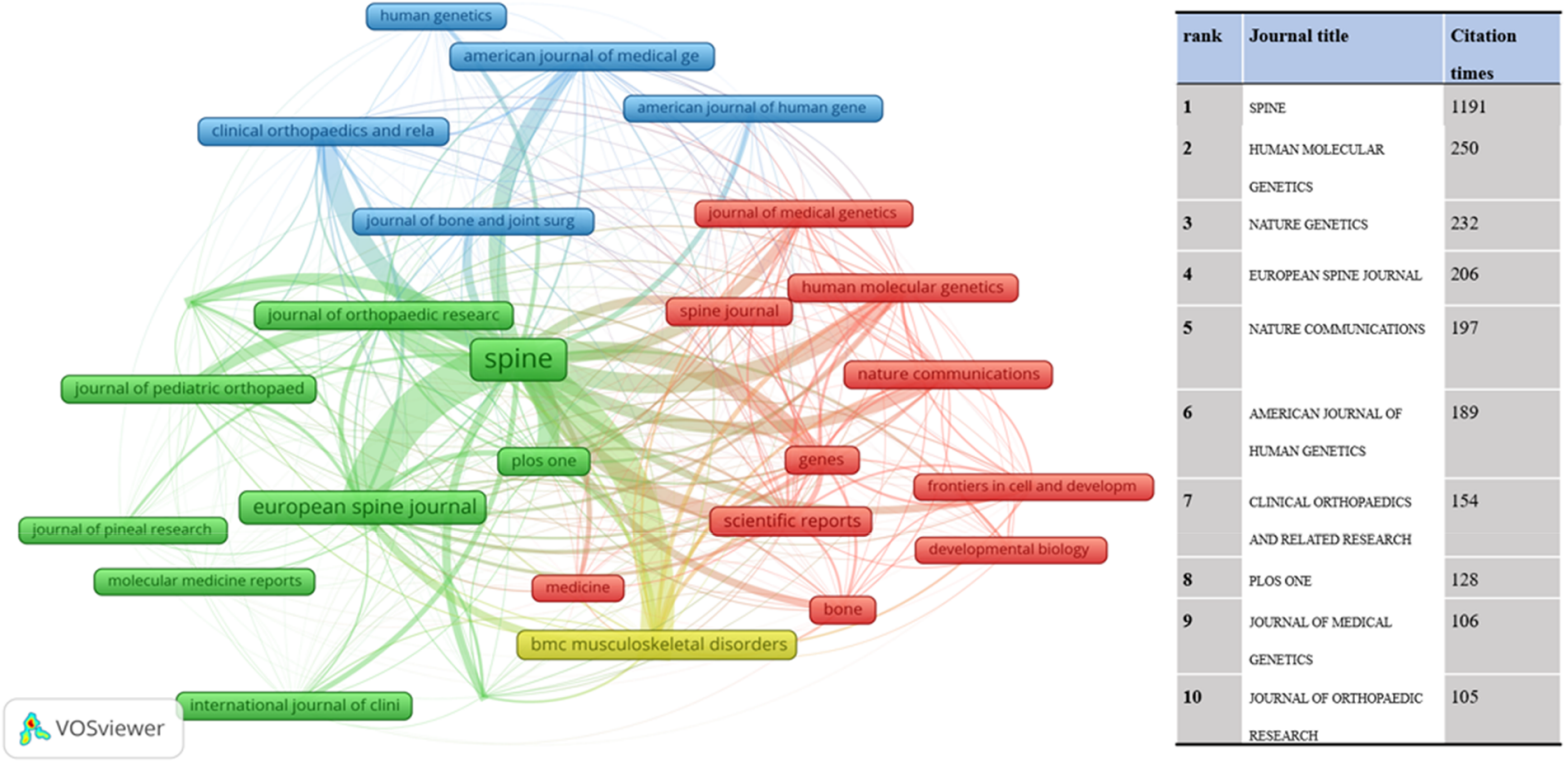
The network map of the journals cited contributed to publications on the idiopathic scoliosis genes from 2002 to 2022.

**Figure 6:**
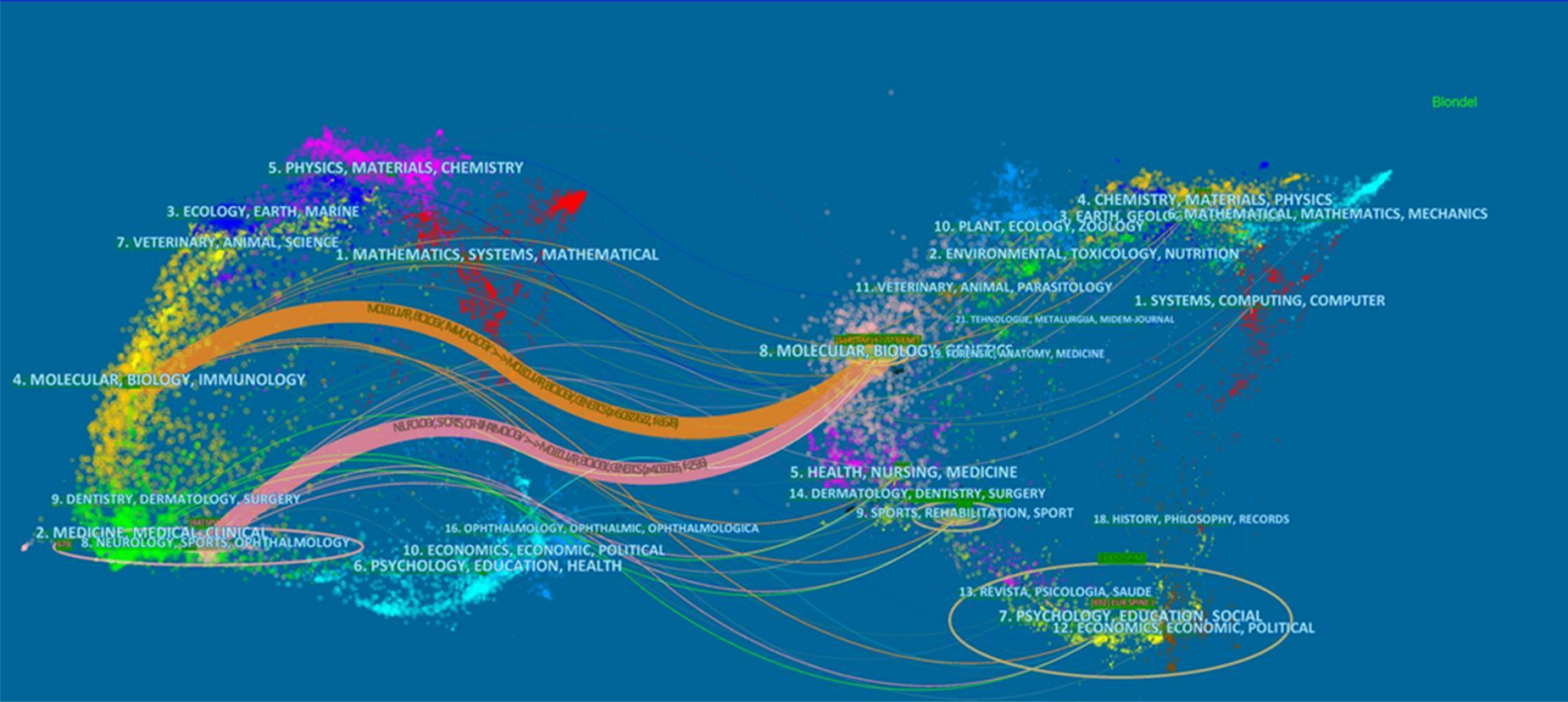
The dual-map overlay of journals contributed to publications on idiopathic scoliosis-genes from 2002 to 2022.

The citation relationship in an academic journal represents the flow of knowledge in this research field. The data of the cited journals display the number of citations received by the journal in the JCR year, which reflects academic status. Table 2 summarizes the details of the top 10 journals cited. The collaborative networks of these journals are revealed in Figure 5. The most cited journal was SPINE (432), followed by BONE JOINT SURG AM (292). The bispectrum overlays of the journals are displayed in Figure 6. The left side shows cited journals, and the right side shows cited journals. The colored paths indicate citation relationships. Molecular/biology/genetics are often cited by molecular/biology/immunology and neurology/sports/ophthalmology (Figure 6).

**Table 2:** Top 10 cited papers.

Rank	Citation name	Pmid	Number of citations	Study results
1	Genetic variants in GPR126 are associated with adolescent idiopathic	23,66,6238	125	This study identified a novel susceptibility locus on chromosome 6q24.1.
2	Genome-wide association study identifies new susceptibility loci for adolescent idiopathic scoliosis in Chinese girls	26,39,4188	107	This paper is the first to use GWAS to study AIS-related gene variants in a Chinese population.
3	Association of estrogen receptor beta gene polymorphisms with susceptibility to adolescent idiopathic scoliosis	19,33,7,134	92	This study examined the association between estrogen receptor beta gene polymorphisms and susceptibility to adolescent idiopathic scoliosis.
4	Genetic association study of insulin-like growth factor-I (IGF-I) gene with curve severity and osteopenia in adolescent idiopathic scoliosis	17,10,8,398	85	This literature explored the connection between two SNPs in the promoter region of the *IGF-I* gene and AIS.
5	Association of *GPR126* gene polymorphism with adolescent idiopathic scoliosis in Chinese populations	25,47,9,386	83	The paper offers evidence for a robust correlation between three SNPs in the intron of the *GPR126* gene and AIS susceptibility.
6	Familial (idiopathic) scoliosis. a family survey	56,41,594	82	This article investigates the prevalence of familial IS in a specific population.
7	Association of estrogen receptor gene polymorphisms with susceptibility to adolescent idiopathic scoliosis	16,64,8,749	78	This study explored the relationship between estrogen receptor gene polymorphisms and the risk of AIS.
8	Understanding genetic factors in idiopathic scoliosis, a complex disease of childhood	19,42,4,484	77	This article provides a review of the genetic causes of idiopathic scoliosis.
9	Localization of susceptibility to familial idiopathic scoliosis	10,98,4,791	77	This paper identifies genes that encode chromosomal loci associated with susceptibility to idiopathic scoliosis by positional cloning.
10	Effects of Bracing in Adolescents with idiopathic scoliosis	24,04,7,455	76	The paper investigates the role of support in the ASI bending process.

### 3.4. Analysis of references

**Figure 7:**
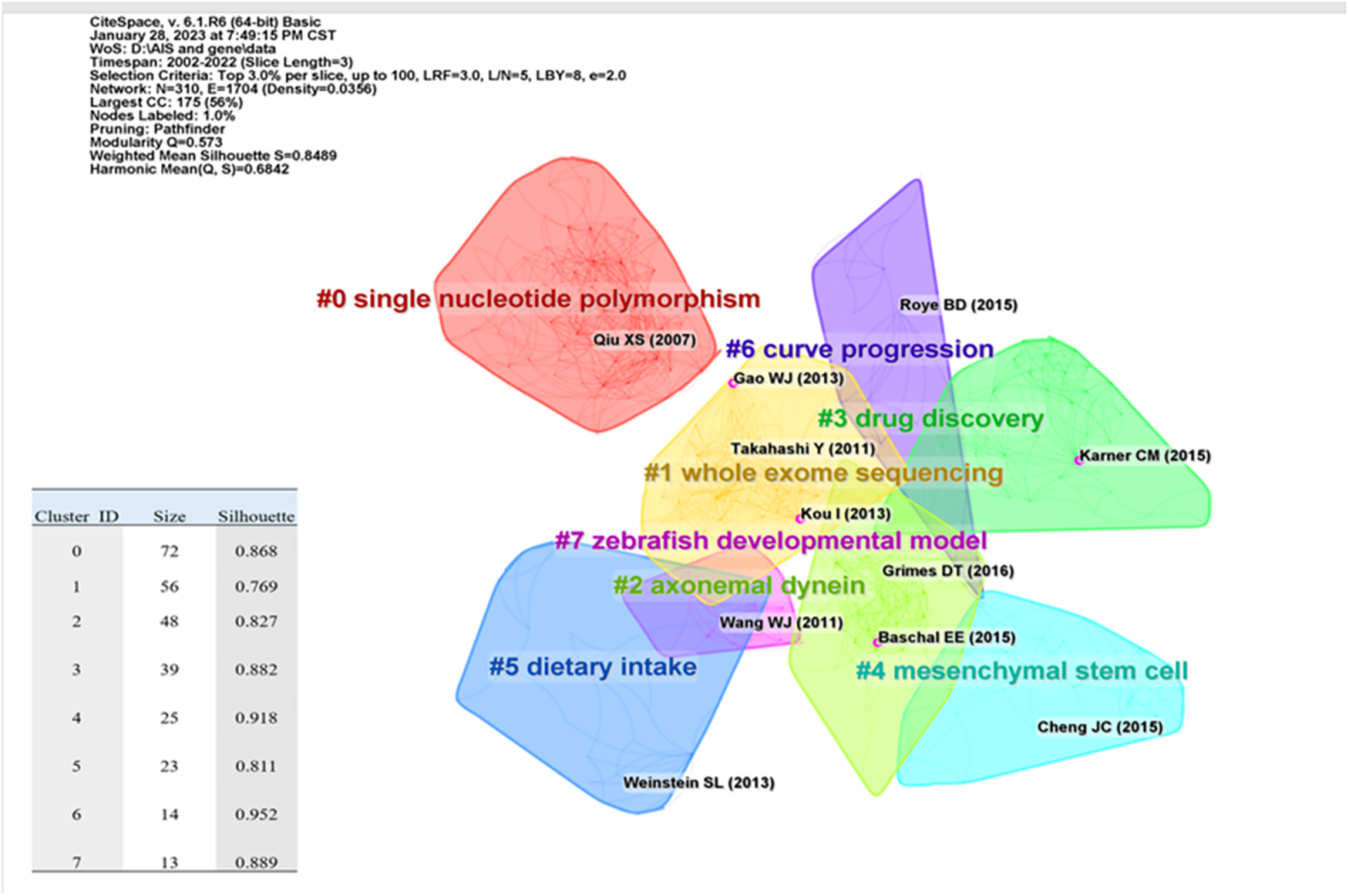
Citation clustering results are shown: the literature in purple circles are articles with high mediated centrality, with mediated centrality > 0.1, representing that the literature plays an essential role in the network and development.

Citing papers constitute the frontiers of knowledge, and cited papers constitute the basis of knowledge. Numerous highly cited articles have been published in top journals on human idiopathic scoliosis genetic studies, including those in Nature and Science. The topics of these articles include genome-wide associations, protein-coupled receptors, biological pathways, and myelination. Reference co-citation refers to the phenomenon in which more than two articles cite the same reference. By analyzing the clusters and critical nodes in the co-citation network, the knowledge structure of the IS gene research field can be revealed (14), as well as the relationship between research themes and subfields (15). The Modularity Q score was 0.573 (> 0.5), indicating that the network was reasonably divided into loosely coupled clusters. The Weighted Mean Silhouette was 0.8489 (> 0.5), and the Harmonic Mean (Q, S) was 0.6842, indicating that the homogeneity of these clusters was acceptable (Figure 7). The largest cluster, # 0, was labeled as "single nucleotide polymorphism"; cluster # 1 was "whole exome sequencing" (9, 16–18), and cluster #2 was "axonemal dynein" (19–21). Cluster # 3 is "drug discovery" (3, 22, 23). Cluster #4 is "mesenchymal stem cell" (24–26). Cluster # 5 was "dietary intake" (27–29). Cluster # 6 was the "curve progression" (30–32), and cluster # 7 was the "zebrafish developmental model" (33, 34). The most cited references in each cluster were color-coded and shown, including those with the strongest mediation centrality (Figure 7).

### 3.5. Keywords

**Figure 8:**
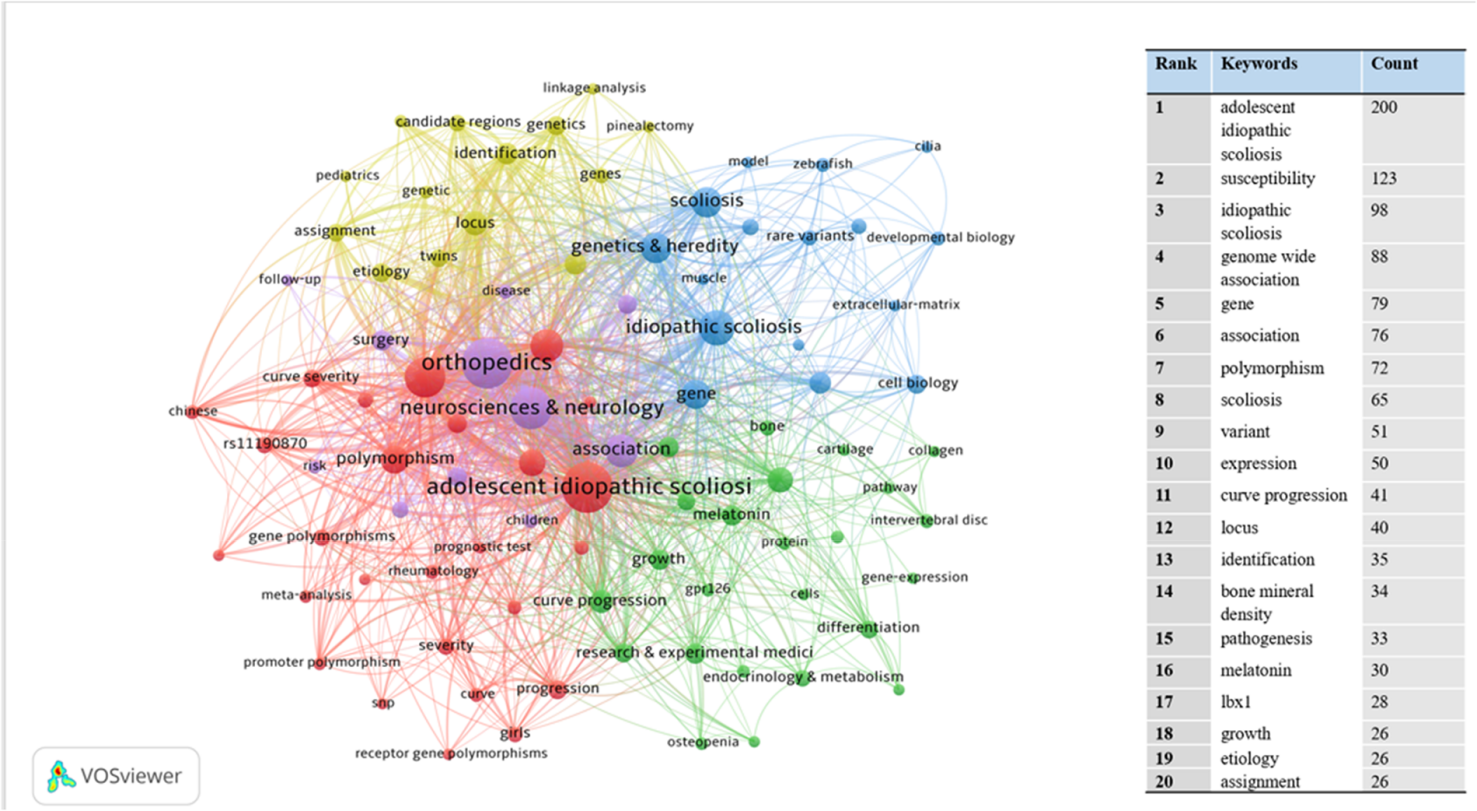
The network map of key worlds contributed to publications on idiopathic scoliosis-genes from 2002 to 2022. The co-occurrence of high-frequency keywords represents the focus and research trend.

**Figure 9:**
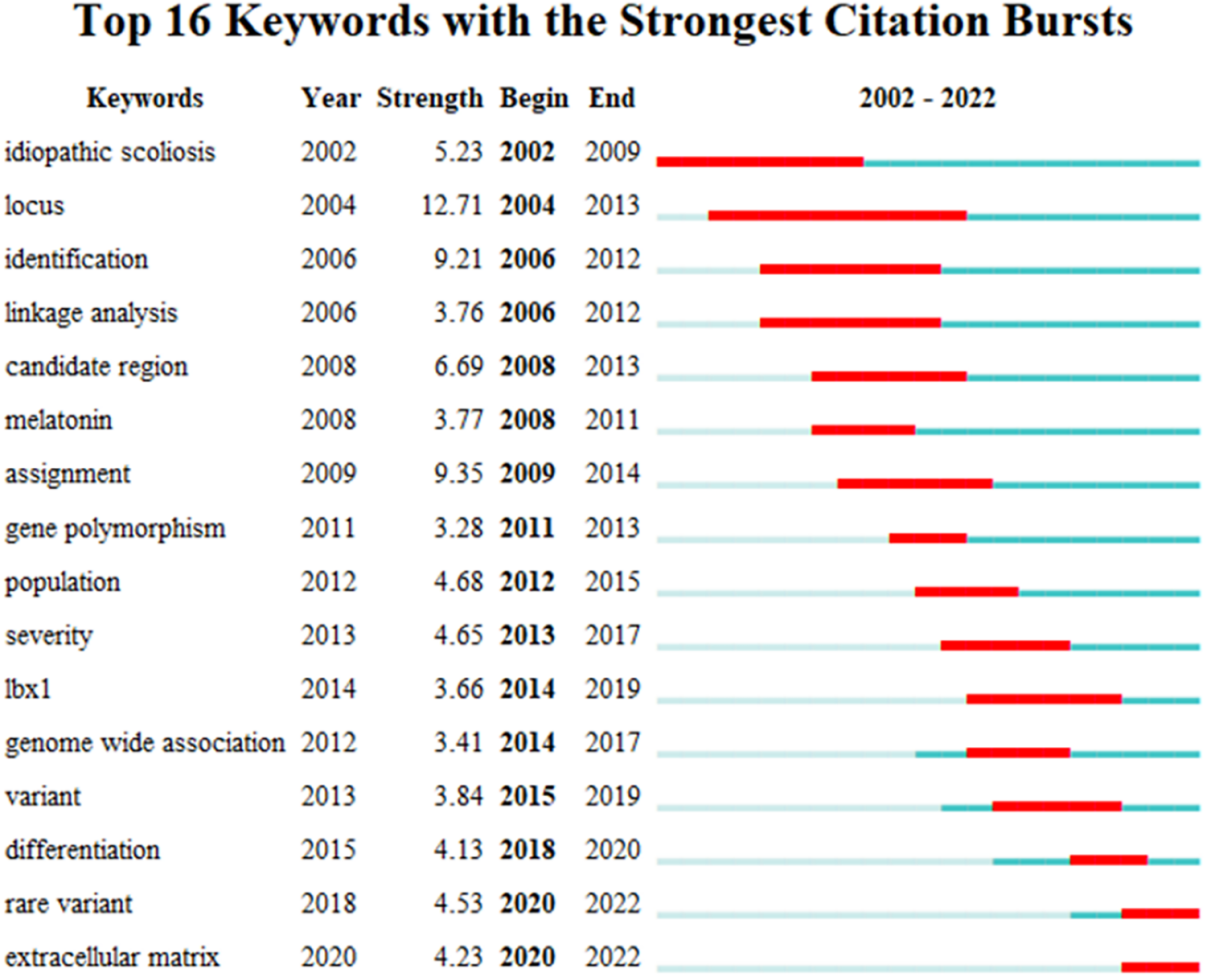
The left side displays the burgeoning keywords, while the right side displays the hot spot transfer trend. The red bars reflect that researchers placed high importance on this keyword at this time.

We extracted and analyzed keywords from the relevant literature. The top 16 keywords with the highest number of occurrences were listed (Figure 8). In addition to idiopathic scoliosis, susceptibility and genome-wide associations appeared more than 80 times. We performed an emergent analysis of keywords and analyzed the temporal trends of hotspot shifts based on the top 16 emergent keywords, such as rare variant (2018–2022) and extracellular matrix (2020–2022) (Figure 9).

## 4. Discussion

### 4.1. Knowledge map of references

Previous research has led to a better organizational map of genetic studies on idiopathic scoliosis (Figure 7). Based on the analysis of references, we analyzed the knowledge base, study objectives, and potential research directions in this research field.

In cluster 0, studies have shown that single nucleotide polymorphisms (SNPs) in the gene promoter region may be necessary to identify IS susceptibility genes. Multiple AIS susceptibility genes, including *LBX1*, *GPR126*, *BNC2*, *PAX1*, *LBX1-as1*, *BCL2*, *AJAP1*, *PAX3*, *TNIK*, *MEIS1*, *MAGI1*, *TGFB1*, *MIR4300HG*, and *TPH1* genes, have already been identified in Caucasian, Japanese, and Chinese populations (35, 36). However, most of these findings are difficult to replicate in multi-ethnic populations. Recent advances in IS etiology research have focused on bone morphogenetic protein (*BMP4*), interleukin (IL6), leptin, matrix metalloproteinase 3 (*MMP3*), chromosome domain caspase DNA DNA-binding protein 7 (*CHD7*) (8), and promoter polymorphisms of the melatonin 1 B receptor (*MTNR1B*) promoter (35). Furthermore, these genes do not have the same impact on the etiology of IS. There is a great deal of interaction between them, and they are closely coupled (37). Qiu. et al. (29) revealed that MTNR1B gene polymorphism is linked to adolescent idiopathic scoliosis susceptibility (AIS). Subsequent research (36, 38) has revealed that *MTNR1A* gene promoter polymorphism may not be related to the occurrence of AIS or the severity of the curve. Nikolova ST et al. (39) reported that combining *IL-6* and *MMP3* genotypes might be associated with IS susceptibility. The biological effects of IS susceptibility gene polymorphisms could explain the above occurrence, highlighting the importance of verifying gene interactions in IS gene research (40).

Currently, there is no conclusive evidence whether AIS is a single disease or a collection of multiple diseases. Research suggests that AIS may be influenced by genetic factors, environmental factors, and spinal growth and development (41–44). Additionally, a significant body of research indicates the presence of diverse morphological features, clinical presentations, and prognoses among AIS patients (45). Complexity and heterogeneity are key characteristics of AIS etiology and phenotype, suggesting that AIS can be considered as a relatively complex group of diseases.

In cluster 1, "whole exome sequencing," which is limited by the low efficiency of genetic marker definition, gene linkage analysis can only identify chromosomal regions that may contain disease-related genes (46). Methods for obtaining more genetic evidence for the IS gene through high-throughput sequencing have been widely used. Genome-wide association studies (GWAS) have identified potential common risk alleles, especially those located in or near *LBX1* (20, 22) and *GPR126*/*ADGRG6* (3, 47, 48). Whole exome sequencing (WES) studies have found rare variations in extracellular matrix genes that can lead to AIS phenotypes in families and unrelated individuals (28, 49, 50).

Among cluster 2, "axonemal dynein," kinesin family 6 (KIF6) mutants, and PTK7 were reported to be IS candidate genes. Grimes et al. (45) defined the limited development window of motor cilia by investigating the flow characteristics of cerebrospinal fluid (CSF) in a zebrafish model with a PTK7 gene mutation, thus revealing the regulatory mechanism of cilia activity recovery during scoliosis progression. Baschal et al. (20) revealed predictive variants in IS-related genes, static cilia, and other actin-based cell projections using GO enrichment analysis. Functional studies of these variants confirmed the relationship between IS and these variations. However, the current research in this area is limited, which calls for further investigation and verification of the causes of the disease to establish a foundation for its prevention and treatment.

In cluster 3,"drug discovery,"Karner et al. (51) demonstrated a *GPR126* mechanism of action in axial cartilage, indicating that GPR126cAMP agonists could still repair the decrease in *GPR126* signaling in cartilage cells, which might be a valuable resource for drug exposure in IS. Currently, this type of medication has demonstrated significant potential in the treatment of idiopathic scoliosis. Therefore, it may be considered as a promising candidate in the development of drugs for both preventing and treating idiopathic scoliosis.

In cluster 4, "mesenchymal stem cells," irregular bone development and decreased bone mineral density were initially discovered as bone metabolism problems in patients with AIS. Bone marrow mesenchymal stem cells (BMMSCs) have been studied for their ability to promote osteogenic differentiation and proliferation (52) to improve bone mass homeostasis (53).

BMMSCs use miRNAs for several biological activities, including transmembrane transport, cytokinesis, DNA-dependent transcription, GTPase-mediated signal transduction, and response to hypoxia. Hui SYet al. (54) discovered 54 previously unknown differentially expressed genes in AIS patients with BMMSCs, and identified the differential expression profiles of miRNAs and related pathways, providing new insights into the pathophysiology of AIS and the mechanism of bone loss.

In Cluster 5, "dietary intake". The dietary mechanisms underlying IS have been postulated to involve several hypotheses, including DNA methylation (55, 56), lipid peroxidation (57), and neurodevelopmental processes (58). As an influential environmental factor (59), diet exerts a certain influence on the patterns of methylated cytosines ("methylome") during childhood growth and development, ultimately impacting gene expression patterns (60). Dietary habits exert regulatory control over the genetic phenotype plasticity of idiopathic scoliosis during the developmental plasticity window through intricate gene regulatory mechanisms (62). Keiko Asakura et al. (63) investigated the relationship between dietary habits and IS in 2,431 subjects, 47.8% of whom had AIS, and found no significant association between dietary habits and AIS. In contrast, research (64) has discovered a correlation between dietary intake and IS in children. Several studies have reported that adolescents diagnosed with idiopathic scoliosis display notable variations in anthropometric measurements, such as increased height, reduced body mass index, and decreased bone density, compared to their age-matched counterparts (65). Daily supplementation of calcium and vitamin D has been shown to enhance the trabecular bone area, density, and strain index at the distal end of the tibia and radius. Additionally, it has been found to increase the cortical area of the tibial shaft (66). The cumulative effect of dietary imbalances across multiple generations in both parents has resulted in variations in 1C genes, related epigenetic regulatory factors, and methylation patterns of target DNA sequences (57). The metabolic adaptations induced by traditional dietary habits within a family can give rise to significant transgenerational effects (67). It is worth noting that the asymmetry of paraspinal muscles in AIS patients involves alterations in both chronomics and the genome (68, 69). These alterations are potentially associated with lipid peroxidation. Additionally, research suggests that dietary antioxidants may have the potential to mitigate the process of lipid peroxidation (58). Moreover, scientific investigations have revealed a noteworthy correlation (55) between enhanced dietary quality and the predictive factors pertaining to the accelerated aging epigenetic clock. For example, it has been observed that elevated fructose consumption induces DNA methylation in the promoter region of CPT1A, while a high-fat diet exerts an inhibitory effect on this methylation process, thereby modulating the organism's energy metabolism levels (70). While the precise molecular mechanisms underlying the impact of diet on the genetic phenotype of IS have yet to be fully elucidated, it is evident that diet, as a specific environmental factor, plays a conspicuous role in the epigenetic alterations associated with IS (59). Maintaining good dietary habits and paying attention to what we eat may be helpful in reducing the incidence of idiopathic scoliosis.

In cluster 6, which focuses on "curve progression," accurate early diagnosis and precise prediction of curve progression are crucial in mitigating the long-term adverse effects associated with AIS treatment in the management of AIS (71). Age, gender, skeletal maturity, Tanner stage, and menarche were frequently used clinical variables to identify people at risk of curve progression (72). Simultaneously, identifying the non-genetic basis of the risk of curve progression has emerged as a research hotspot. Roye BD (73) et al. Identified epigenetic differences in IS twins and genomic variations between individuals with distinct curves, clarifying that non-genetic factors must be involved in curve progression in AIS patients. Meng YC et al. (74) affirmed that epigenetic and genetic markers could be used as potential predictors of curve progression. However, the majority of gene tests associated with it have proven to be unsuccessful. The potential reasons for such outcomes may be attributed to the complex and dynamic nature of epigenetics. The emergence of epigenetics has provided new insights into AIS research (55). Epigenetic mechanisms allow organisms to respond to environmental changes through alterations in gene expression, maintaining gene silencing or activation through chemical modifications of DNA regions and protein modifications during cell development (75). As a disease with unclear pathogenesis, despite the failures of most gene tests associated with IS, the reasons behind these results may be attributed to the complex and dynamic nature of epigenetics, which is influenced by multiple factors (61). These factors include, but are not limited to, the growth and development window (41), lifestyle (59), temperature (76), geographical latitude (77), which may explain the test failures.

Despite genetic variability and a lack of suitable developmental models, little is known about the genesis and mechanism of idiopathic scoliosis in cluster 7, "zebrafish developmental model." Numerous genes with similar expression features exist in both humans and zebrafish. Influenced by genomic perturbation, bio-imaging, and high-throughput small-molecule screening, zebrafish are naturally sensitive to spinal curvature and can reasonably accept vigorous positive gene screening, hence providing strong support for the pathogenicity study of human IS-related gene mutations. By combining zebrafish IS models with high-throughput small-molecule screening, advanced genome-editing tools enable robust precision medical services for IS investigation.

### 4.2. Research hotspots and frontiers

Keywords can express existing research questions or concepts intensively, whereas burst keywords can reflect emerging trends and research frontiers in this field. The two frontiers of relevant research are as follows: extracellular matrix (2020–2022) and rare variant (2018–2022).

#### 4.2.1. Extracellular matrix

Long-term deformation and changes in mechanical load on scoliosis intervertebral disc tissue affect cells in several ways. As determined by gene expression analyses using cDNA arrays and RT-PCR, extracellular matrix components, such as polymerins, contain large quantities of mRNA (78). In contrast to physiological aging, morphological disc degeneration in scoliosis is accelerated by approximately 22 years (79). In response to changes in the mechanical environment of the tissue, patients with scoliosis synthesize a more robust metabolic matrix at a young age (80).

Spinal developmental disorders increase the risk of neurological and other physiological dysfunctions in adolescents with scoliosis. The structural integrity of the extracellular matrix and the variation in homeostasis factors, for example, have a non-negligible impact on human cartilage biogenesis and intervertebral disc development, which may be one of the causes of AIS (81). Furthermore, studies (82) revealed that MAPK7 has a variety of functions in cell migration and proliferation, and osteoblast abnormalities caused by MAPK7 variants have been implicated in zebrafish and human IS.

#### 4.2.2. Rare variant

Numerous AIS candidate genes have been identified using high-throughput sequencing techniques in candidate gene association studies. The validity of these candidate genes has been questioned because there is insufficient evidence that they can frequently be replicated in various populations (46). As rare genotypes are unexpectedly identified in the general population, it is generally assumed that rare variants have a more significant impact on the genetics of complex diseases than common variants. Patten SA et al. (83) reported three rare variants of *POC5* that are related to AIS function using WES. Subsequently, rare variants of *HSPG2* were reported as potential factors for susceptibility to AIS in Caucasians (49). Haller G et al. (84) reported that rare variants in extracellular matrix genes substantially affect the risk of AIS in patients of European ancestry. In a recent study, Xia C et al. (85) validated the effect of the rare variant *HSPG2* on IS progression. Overall, IS is a complex disease, and it is worthwhile to identify and validate rare variants associated with IS and investigate their impact on patient phenotypes.

### 4.3. Limitations

Although the highly regarded WoSCC database was utilized in this study to identify the knowledge map and hotspots of IS gene research, there are crucial limitations that must be considered. Firstly, WoSCC only includes certain journals and publications, resulting in limited coverage and an inability to represent all subject areas. Secondly, WoSCC largely concentrates on English-language literature, potentially overlooking non-English language literature that could greatly influence idiopathic scoliosis-related gene research. Additionally, the search time frame may not be extensive enough to fully capture short-term research trends. Finally, while bibliometric analysis constitutes a relatively objective method for analyzing literature, authors, and topics through network science, machine learning, among others, these methodologies also possess their own limitations. For instance, keyword-based analysis may disregard semantically complex literature, leading to inaccuracies in analysis results. Moreover, this study failed to comprehensively evaluate some large cohort studies or specific cases, potentially missing cutting-edge discoveries such as new IS gene identification. The functional annotation and enrichment analysis of well-known IS genes might not have been administered to tools like DAVID, thereby possibly constraining the discovery of new mechanisms of IS disease. Hence, future research needs to continue refining databases, technologies, and methodologies to thoroughly and expansively explore the forefront of IS gene research.

## 5. Conclusions

This study provides a visual analysis and review of the IS gene-related literature using a bibliometric analysis method. In addition to identifying current research hotspots, the report provides an objective and detailed overview of the research found over the last two decades. Furthermore, it envisions a future development model for the IS gene field, which can be a reference for scholars and clinicians engaged in clinical research and treatment in this field.

The level of interest in IS-related gene analysis is higher and higher in recent years. Extracellular matrices and rare variants are becoming the hotspots and frontiers of research. Meanwhile, early prevention and treatment of sensitive groups were positively affected by identifying molecular markers in IS development. The discovery of recovery patterns of ciliary motility can lead to the development of new therapeutic agents for IS. Further functional analysis may clarify the downstream pathways of the identified susceptibility genes in the pathogenesis and mechanism of action of IS. Understanding the mechanisms and mutation patterns of the downstream pathways of susceptibility genes in IS pathogenesis could be significantly enhanced by optimizing the functional analysis. In the future, more in-depth studies on IS should be carried out from the aspects of extracellular matrices, rare variants and molecular markers, in order to provide insights for understanding the pathogenesis of IS and the development of anti-IS drugs. Ultimately, and most significantly, it can be argued that the prevention and intervention of curve progression rely predominantly on empirical evidence. Therefore, it is imperative to explore the integration of empirical approaches with epigenetics mechanisms to establish more efficacious preventive measures.
